# Initial evidence of a 50% reduction of contrast media using digital variance angiography in endovascular carotid interventions

**DOI:** 10.1016/j.ejro.2020.100288

**Published:** 2020-11-17

**Authors:** Viktor I. Óriás, Dávid Szöllősi, Marcell Gyánó, Dániel S. Veres, Sándor Nardai, Csaba Csobay-Novák, Balázs Nemes, János P Kiss, Krisztián Szigeti, Szabolcs Osváth, Péter Sótonyi, Zoltán Ruzsa

**Affiliations:** a Kinepict Health Ltd, 1026, Júlia u 11, Budapest, Hungary; b Bács-Kiskun County Hospital, 6000, Nyíri út 38, Kecskemét, Hungary; c The Heart and Vascular Center, Semmelweis University, 1122, Városmajor utca 68, Budapest, Hungary; d Department of Biophysics and Radiation Biology, Semmelweis University, 1094, Tűzoltó u 37-47, Budapest, Hungary

**Keywords:** Digital variance angiography, Digital subtraction angiography, Carotid artery disease, Iodinated contrast media, Dose management, Safety

## Abstract

**Purpose:**

In previous clinical studies Digital Variance Angiography (DVA) provided higher signal-to-noise ratio (SNR) and better image quality than Digital Subtraction Angiography (DSA). Our aim was to investigate whether this quality reserve of DVA provides an opportunity for the reduction of iodinated contrast media (ICM) in carotid X-ray angiography (CXA).

**Method:**

Our prospective study enrolled 26 patients (67.0 ± 8.1 years) undergoing carotid percutaneous transluminal angioplasty. The SNR of DSA and DVA image pairs obtained by a standard (100 %, 6 mL ICM) or a low-dose (50 %, 3 mL ICM) protocol were compared. Visual evaluation of all images was performed by five specialists using a 5-grade rating scale. The quality of DSA_100_ and DVA_50_ videos was also compared.

**Results:**

DVA provided more than two-fold SNR, the median SNR_DVA_/SNR_DSA_ ratio was 2.06 (100 %) and 2.25 (50 %). In the visual evaluation, the DVA_100_ score (3.73 ± 0.06) was significantly higher than the DSA_100_ score (3.52 ± 0.07, Wilcoxon p < 0.001), and the DVA_50_ score (3.64 ± 0.13) was also significantly higher than the DSA_50_ score (3.01 ± 0.17, Wilcoxon p < 0.001). While the low-dose protocol significantly decreased the DSA score (Mann-Whitney p < 0.01, DSA_100_ vs DSA_50_), it had no effect on the DVA score (DVA_100_ vs DVA_50_). There was no statistical difference between the DSA_100_ and DVA_50_ scores. Evaluators preferred the diagnostic value of DVA_50_ to DSA_100_ videos in 61% of comparisons, the interrater agreement was 69 % (Fleiss’ kappa 0.35, p < 0.001)

**Conclusions:**

Our data show that DVA allows a substantial (50 %) ICM reduction in CXA without affecting the quality and diagnostic value of angiograms.

## Highlights

• Digital Variance Angiography (DVA) is a novel medical image processing method.
• DVA provides better image quality than Digital Subtraction Angiography (DSA).
• The quality reserve of DVA allows the reduction of contrast agents in angiography.

## 1 Introduction

Atherosclerosis, the accumulation of sclerotic plaques in the wall of blood vessels, and the concomitant stenosis of arteries is the major etiological factor of cardiovascular disorders, the leading cause of death and disabilities worldwide [1]. The incidence of carotid artery (CA) stenosis, a major risk factor of ischemic stroke is strikingly high in the elderly population (> 65 years), it is estimated in the range of 5–10 % in the US [2]. Thus, the diagnosis and treatment of CA stenosis is of great importance.

For decades Digital Subtraction Angiography (DSA) was the reference standard for examining carotid vessels and neurovascular pathology. Although the appearance of non-invasive imaging techniques, like computed tomography angiography, magnetic resonance angiography [3] or color Doppler ultrasound examination gradually replaced intra-arterial angiography in the diagnostic practice [4,5], DSA remains the method of choice in endovascular CA interventions or when the results of non-invasive methods are not conclusive [6].

DSA records a native image mask that is subtracted from the subsequent contrast-enhanced image series, thereby blood vessels filled with iodinated contrast media (ICM) are clearly visualized, but the irrelevant anatomic structures disappear [7]. Although the introduction of low osmolality contrast agents has substantially decreased the risk of adverse reactions (like allergic skin irritation, anaphylactic shock or renal failure), the incidence of these events is still in the 1–3 % range [8]. The neurological complications (stroke or transient ischemic attacks) of intra-arterial carotid angiography reduced to almost zero in the last decade [9] but contrast-induced nephropathy (CIN) is still an existing problem, especially in patients with impaired renal function [10]. The results of the AMACING study [11] has shown that the occurrence of CIN increased from 2.7 % (patients with normal renal function) to 13.6 % in patients with eGFR <30 mL/min/1.73 m^2^. The reduction of the amount of ICM is a current research topic in medical imaging [12,13], because it is crucial for the safety of patients, however the concomitant decrease of image quality hinders these efforts.

Kinetic imaging was developed to obtain more information from medical examinations using penetrating radiation [14]. The advanced statistical processing of intensity values provides additional information, thereby improves image quality. Application of this principle to angiography led to the development of Digital Variance Angiography (DVA). In contrast to DSA, DVA does not use a mask, but calculates standard deviation, variance and other time-derived parameters of the X-ray attenuation for every pixel in an unsubtracted image series. This algorithm enhances the functional motion-related information (i.e. the flow of contrast agents) but suppresses the noise, therefore the signal-to-noise ratio (SNR) and consequently image quality is greatly improved. Recently the functional and diagnostic capabilities of DVA were evaluated in clinical trials and DVA provided higher SNR and better image quality than DSA in lower limb ICM [15,16] and CO_2_ angiography [17,18]. This quality reserve might be used for the reduction of radiation dose or contrast material amount, therefore DVA might enhance the safety of endovascular interventions.

The aim of the current study was to investigate whether the significant quality reserve of DVA could be converted to ICM reduction, therefore we compared the SNR and image quality of DSA and DVA images and videos obtained during carotid percutaneous transluminal angioplasty (PTA) intervention, using both standard (100 %, 6 mL) and low-dose (50 %, 3 mL) ICM protocol.

## 2 Materials and methods

This study was registered and approved by the National Institute of Pharmacy and Nutrition (reference number OGYÉI/69,206/2017) in Hungary. The protocol was designed in accordance with the standards of the Hungarian Medical Research Council and the Helsinki Declaration. All enrolled patients signed a written informed consent after being verbally informed by a physician.

### 2.1 Patient selection and study design

The prospective study enrolled 26 patients undergoing carotid percutaneous transluminal angioplasty (PTA) between January 2018 and June 2018 at the Bács-Kiskun County Hospital (Kecskemét, Hungary). The mean ± SD age was 67.0 ± 8.1 years (23 males 67.3 ± 8.1 years, 3 females 64.7 ± 9.8 years). Table 1 shows the detailed demographic data. The inclusion criteria were specialist referral for the procedure and a glomerular filtration rate over 60 mL/min /1.73 m^2^. The exclusion criteria were severe heart or respiratory failure, glomerular filtration rate under 60 mL/min /1.73 m^2^ or known iodine sensitivity/allergy. Patient enrolment ended when we reached the number of 26 patients. The desired number of patients was planned by the recommendation of the United States Food & Drug Administration [19]. All patients were scheduled for the intervention independently from our study, based on the opinion of a referring vascular surgeon, angiologist or neurologist.

**Table 1:** Detailed demographic data of the study.

		Age (years)	Height (cm)	Weight (kg)	BMI
All (26)	Mean	67.0	176	85.4	27.1
	SD	8.1	5	14.5	3.9
Female (3)	Mean	64.7	169	74.0	25.9
	SD	9.8	2	9.8	3.6
Male (23)	Mean	67.3	177	86.9	27.7
	SD	8.1	4	14.5	4.0

Abbreviations: SD: standard deviation, BMI: body mass index.

**Fig. 1:**
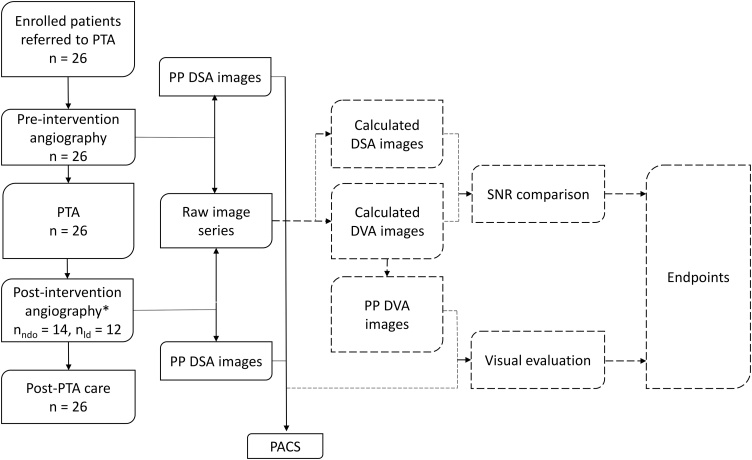
Flowchart of the study. Solid lines represent institutional standard-of-care, while dashed lines represent study protocol. * Low-dose protocol was applied only in post-intervention angiography in 12 patients and 19 runs. No patient received more than 100 mL contrast media throughout the whole procedure. PTA: percutaneous transluminal angioplasty, PP: postprocessed, DSA: Digital Subtraction Angiography, DVA: Digital Variance Angiography, PACS: Picture Archiving and Communication System, SNR: Signal-to-Noise Ratio, n_ndo_: number of ‘normal dose only patients’, n_ld_: number of low dose patients.

Our endpoints were to determine whether DVA or DSA provided higher SNR and better subjective image quality. Both DVA and DSA images used for SNR calculation were generated using raw image data obtained from the angiography system. The subjective image quality of DVA and DSA images and videos was evaluated using randomized online forms. Fig. 1 shows the detailed study design.

### 2.2 Image acquisition

All patients received subcutaneous infiltration anesthesia with lidocaine before the arterial puncture. The vascular access point was the distal radial artery. All punctures were performed with ultrasound guidance. After successful arterial puncture, a standard 5-French radial sheath was placed in the radial artery with Seldinger technique. A 5-French Simmons catheter was used for the cannulation of the common carotid arteries. We obtained angiograms from anteroposterior and lateral views of the head from both common carotid arteries. A GE Innova IGS 530 (GE Healthcare) angiography system was used for image acquisition with a low X-ray dose factory DSA preset (4 fps, avg. tube current 280 mA, avg. tube voltage 105 kV, pulse width 85 ms).

An ACIST CVi (ACIST Europe BV) automated contrast injector was used for ICM injection. The standard injection protocol was a 6-ml bolus of the low osmolarity Xenetix350 mg iodine/mL (Iobitridol, Guerbet LLC) nonionic water-soluble contrast agent with 0.5 s rise time and 3 mL/s flow. After the first 14 patients were included in the study and the image quality advantage of DVA was verified, we started using a low-dose protocol with a 3-ml bolus of the same contrast agent with 0.2 s rise time and same flow. Depending on the injected amount of contrast agent during the intervention, either one of the control angiograms or both of them were performed with the standard and the low-dose protocol as well. This way we obtained 19 images with low-dose protocol. No patient received more than 100 mL of contrast agent throughout the whole procedure. The procedures were done by an interventionist with professional experience of over 15 years.

### 2.3 Image processing and post-processing

For the SNR measurements DSA images were calculated based on standard mask subtraction and summation method of the obtained raw image data [20,21]. DVA images were generated retrospectively by the Kinepict Medical Imaging Tool v2.0 (Kinepict Health Ltd) from raw image data obtained from the angiography system according to the algorithm described earlier [15]. Method-specific post-processing features (brightness/contrast adjustment and pixel shift) offered by the Kinepict Medical Imaging Tool v2.0 were applied to reach the best image quality. In this study, retrospective image analysis was applied, but the CE marked, platform-independent stand-alone software can be integrated into the angiography system for real-time operation as well [18], when the DSA and DVA images are displayed simultaneously on the operating room monitor during the intervention

For the visual evaluation post-processed DSA images were produced and saved using the GE Innova workstation. Post-processing features, such as peak opacification, noise filtering and „PixelShift” motion correction were used to obtain the best DSA image quality offered by the angiography system.

### 2.4 Signal-to-noise analysis and comparison

Vascular and perivascular regions of interest (ROIs) were selected manually. The signal amplitude was defined as the absolute difference between the vascular ROIs and their corresponding background ROIs. The noise was considered as the standard deviation of the pixel values of background ROIs. SNR was determined by the ratio of the average signal amplitude and the noise of background [15]. The ratio (R) of SNR_DVA_/SNR_DSA_ was determined to characterize the quality difference between the images. Medians were used for the statistical description of datasets because of the asymmetric distribution of values.

Matlab 2016a (The MathWorks Inc.) and Image J (v. 2.0.0-rc-68/1.52e, Creative Common License, NIH) [22] were used to generate DVA and DSA images, selecting ROIs, and measuring signal and noise. SNRs and their ratios (R) were calculated by Microsoft Excel 2016 (Microsoft).

### 2.5 Visual performance evaluation

A blinded, randomized evaluation of images was carried out by one vascular surgeon, four interventional radiologists and one interventional neuroradiologist with at least 5 years of experience in their field working at two different clinical sites. The images were evaluated in a randomized order. Each image was evaluated once by every participant. 246 images (123 DVA and 123 DSA) were evaluated by five evaluators according to a 5-grade rating scale described below:

  1 poor image quality, unsuitable for diagnosis
  2 low image quality, main vessels are distinguishable but not examinable, unsuitable for diagnosis
  3 medium image quality, the main vessels are examinable, but diagnosis of supracarotid vessels are questionable
  4 good image quality, even supracarotid vessels are examinable, suitable for everyday use
  5 outstanding image quality, much richer in details compared to the everyday routine”

In order to verify the usefulness of reduced ICM protocol in the clinical practice, the quality of 100 % ICM DSA videos were compared to that of 50 % ICM DVA videos (19 pairs). taken from the same patient and direction. The raters had to select the better video in a randomized blinded manner from runs by answering the following question: ‘Which video is more suitable for diagnostic examination?’

### 2.6 Statistical analysis

Analysis of the SNR, along with calculations of SNR medians and confidence intervals were done using Microsoft Excel 2016 (Microsoft, Redmond, WA).

For individual evaluation of DSA and DVA images we calculated the mean and standard error of mean (SEM) of scores given by the raters. Since the distribution was not Gaussian in certain groups, the median and the interquartile range was also determined. Wilcoxon signed rank test (DSA vs DVA) or Mann-Whitney’s *U* test (100 % vs 50 %) was used to compare results in each region and the Kendall’s W was calculated to describe interrater agreement. Calculations for all visual evaluations were made using Stata 15.0 statistical data analysis software (StataCorp, College Station).

For the comparison of videos, the quality score was calculated as the mean percentage of raters choosing the 50 % ICM DVA run over the 100 % ICM DSA run for every corresponding video pair. To describe agreement between raters, percent agreement and Fleiss’ kappa was calculated.

## 3 Results

### 3.1 Signal-to-noise ratio measurement and comparison

SNRs of DVA and DSA images were calculated in a total of 3074 manually selected ROIs using 124 image pairs. The R values of SNR_DVA_/SNR_DSA_ were calculated for each ROI pairs. The distribution of this parameter was strongly asymmetric, therefore the medians and the first and third quartiles (in parentheses) are reported. DVA provided consistently higher SNR values than DSA (Table 2). The R values of SNR_DVA_/SNR_DSA_ were 2.06 (1.58−2.71) and 2.25 (1.66−2.89) for the DVA/DSA image pairs obtained with the standard (100 % ICM) and the low-dose (50 % ICM) protocol, respectively.

**Table 2:** Summary of Signal-to-Noise Ratio measurements.

		SNR_DSA_	SNR_DVA_	SNR_DVA_/SNR_DSA_
Standard protocol	Median	5.41	10.95	2.06
	Q1-Q3	3.23−8.83	6.93−17.51	1.58−2.71
Low-dose protocol	Median	4.00	9.13	2.25
	Q1-Q3	2.61−6.11	5.60−14.41	1.66−2.89

The table shows the median and the first and third quartiles (Q1-Q3) of the SNR values obtained with the different image processing methods and ICM protocols. Standard protocol: 100 % ICM, low-dose protocol: 50 % ICM. Abbreviations: SNR: Signal-to-Noise Ratio; ICM: Iodinated Contrast Media; DSA: Digital Subtraction Angiography; DVA: Digital Variance Angiography.

### 3.2 Visual evaluations

#### 3.2.1 Single image evaluation

**Fig. 2:**
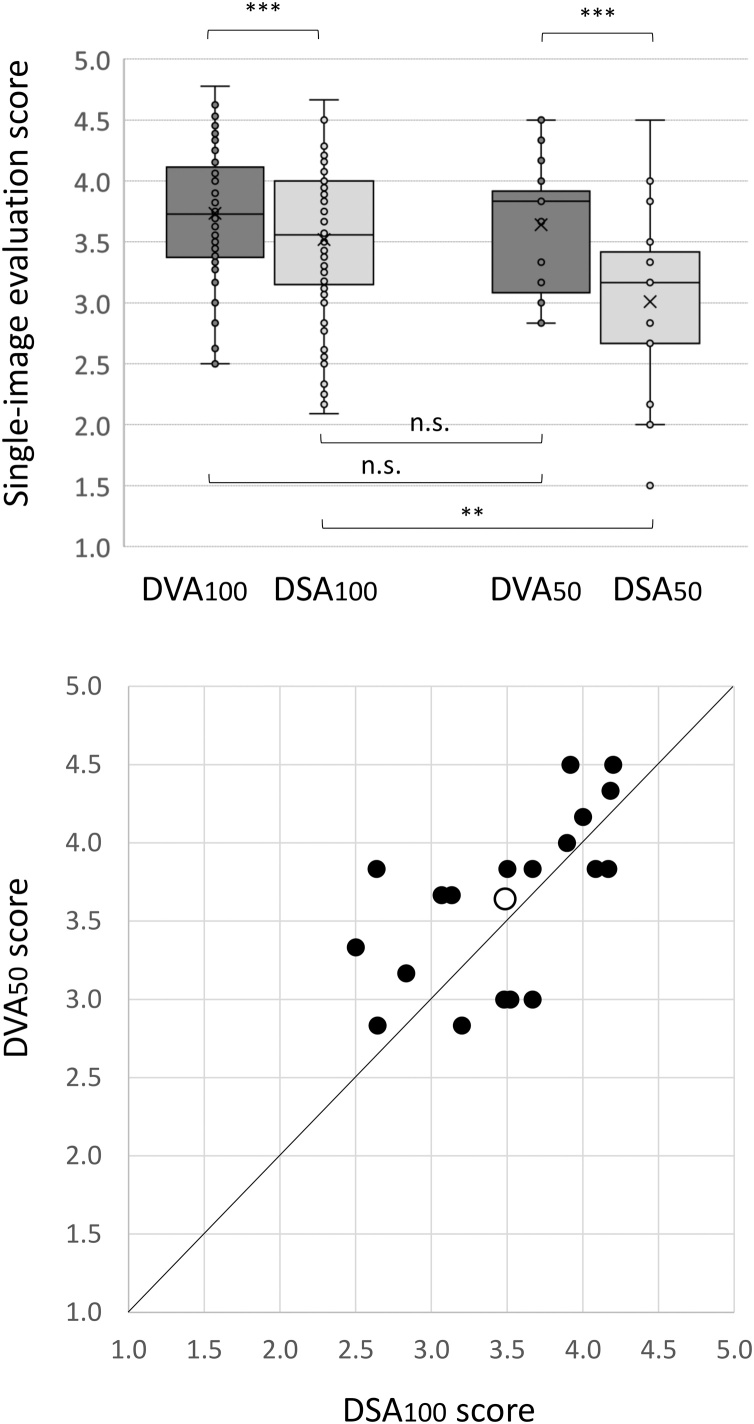
Comparison of single-image evaluation scores. The box and whisker plot (upper panel) shows the median (line), the mean (x) and the interquartile range (box) of each group. Data were analysed by the Wilcoxon signed rank test (comparisons above the boxes) or by the Mann-Whitney *U* test (comparisons below the boxes), depending on the data structure (** p < 0.01, *** p < 0.001). Lower panel: Each point represents a repeated measurement obtained in the same position from the same patient using the normal and the low-dose protocol. The abscissa and the ordinate represent the mean of the DSA_100_ and DVA_50_ scores, respectively. The open circle shows the mean values of the 19 images. Abbreviations: DSA: Digital Subtraction Angiography; DVA: Digital Variance Angiography, n.s. not significant.

The six evaluators rated 246 DSA or DVA images in a blind and randomized manner using a 5-grade rating scale (see Materials and Methods section). The image set contained 104 DSA_100_, 104 DVA_100_, 19 DSA_50_ and 19 DVA_50_ images (the subscript refers to the ICM dose used), thus the number of DSA and DVA images were equal (123 each). The mean ± SEM and the median and interquartile range were calculated for each group and the data were statistically analysed by the Wilcoxon signed rank test (for the DSA vs DVA comparisons) or the Mann Whitney *U* test (for the 100 % vs 50 % comparisons). (Table 3). DVA outperformed DSA in all comparisons. The DVA_100_ score (3.73 ± 0.06, n = 104) was significantly higher than the DSA_100_ score (3.52 ± 0.07, p < 0.001, n = 104), and the DVA_50_ score (3.64 ± 0.13, n = 19) was also significantly higher than the DSA_50_ score 3.01 ± 0.17, p < 0.001, n = 19) (Fig. 2, upper panel).

**Table 3:** Statistical analysis of single image evaluation data.

Image	n	Mean	SEM	Median	Q1-Q3	Wilcoxon	Mann-Whitney U
DVA_100_	104	3.73	0.05	3.73	3.37−4.11	p < 0.001 vs DSA_100_	p = 0.56 vs DVA_50_
DSA_100_	104	3.52	0.05	3.56	3.15−4.00		p = 0.52 vs DVA_50_
DVA_50_	19	3.64	0.13	3.83	3.08−3.92	p < 0.001 vs DSA_50_	
DSA_50_	19	3.01	0.17	3.17	2.66−3.50		p < 0.01 vs DSA_100_

The corresponding DVA-DSA image pairs were analysed by the Wilcoxon signed rank test, groups with different number of elements were compared by the Mann-Whitney *U* test. Abbreviations: n: number of elements, SEM: standard error of mean, Q1-Q3: interquartile range first and third quartile. DSA: Digital Subtraction Angiography; DVA: Digital Variance Angiography. _100_: Normal dose protocol (6 mL), _50_: low-dose protocol (3 mL).

**Fig. 3:**
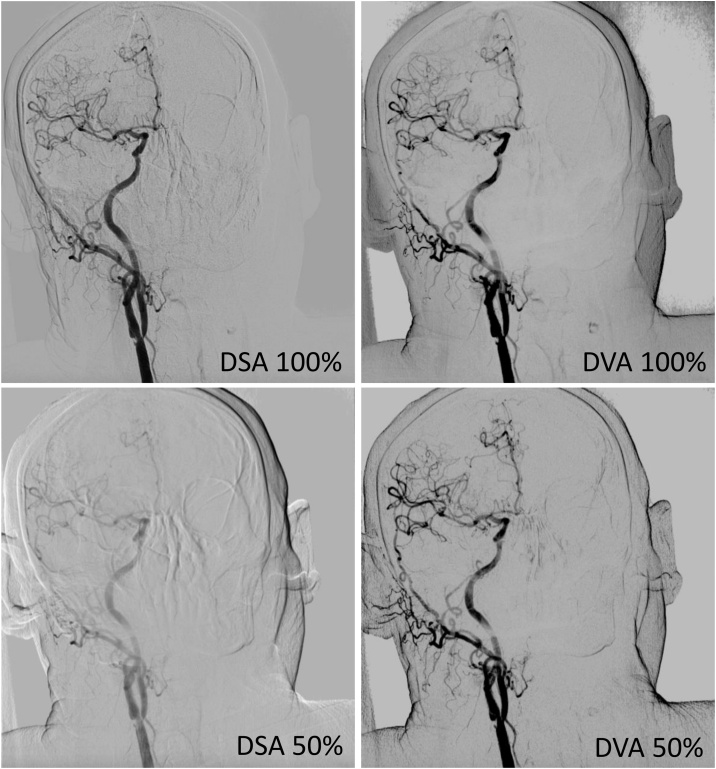
Comparison of representative images obtained with the standard (100 %, 6 mL ICM) and low-dose (50 %, 3 mL ICM) protocols. Images were taken of the same patient and same direction in two runs. All available image quality enhancement techniques (like PixelShift or noise filtering) were applied and brightness and contrast settings were equalized for all 4 images. Abbreviations: DSA: Digital Subtraction Angiography; DVA: Digital Variance Angiography.

While the low-dose protocol significantly decreased the DSA_50_ score (p < 0.01 compared to DSA_100_), it had no effect on the DVA_50_ score that was statistically not different from the DVA_100_ score. The most important finding is that there was no statistical difference between the DVA_50_ and the DSA_100_ score (Fig. 2, upper panel), and in direct comparison of the corresponding means obtained from the same patients, 68 % the DVA_50_ scores was higher than the corresponding DSA_100_ score (Fig. 2, lower panel). The interrater agreement was characterized by the Kendall W calculation. The W values were 0.62, 0.73, 0.50 and 0.50 for the DSA_100_, DSA_50_, DVA_100_, and DVA_50_ image sets, respectively (p < 0.001 in all cases). Fig. 3 illustrates the image quality in the four groups by showing representative images of the same area from the same patient.

#### 3.2.2 Comparison of DSA and DVA videos

To address the quality and clinical usefulness of low-dose protocol DVA, 19 pairs of DSA_100_ and DVA_50_ runs taken from the same patients and direction were compared in a randomized blind manner. The evaluators judged DVA_50_ videos more suitable for diagnostic examination in 61 % of comparisons. The interrater agreement was 69 %, Fleiss Kappa value was 0.35 (p < 0.001). Representative video pairs are available online (see Supplementary material ‘video comparisons’ file).

## 4 Discussion

Dose management efforts are in the focus of medical imaging technologies applying radiation [23] and contrast media [12,13]. Our paper is the first report on the dose management capabilities of the recently developed DVA technology. In this study our aim was to investigate, whether the previously observed quality reserve of DVA allows ICM reduction in interventional carotid artery X-ray angiography, therefore we compared DSA and DVA images and videos obtained with standard (100 % ICM) or low-dose (50 % ICM) protocol. Our data showed that DVA provided an at least two-fold increase in the SNR (Table 2), independently of the ICM dose applied. In line with the results of previous clinical studies, in the randomized and blinded qualitative evaluation DVA images reached significantly higher score than the corresponding DSA images (Fig. 2, upper panel), again in both protocols. While the reduction of ICM dose significantly impaired the image quality of DSA, it had no effect on DVA, that is the quality of DVA_50_ and DVA_100_ was identical. The quality score of DVA_50_ images was higher than that of DSA_100_ images for more than two-thirds of the image pairs (Fig. 2, lower panel), suggesting that DVA in the low-dose protocol performs at least as well as DSA in the standard protocol (Fig. 3). To further substantiate this conclusion, we have compared the quality and clinical usefulness of DSA_100_ and DVA_50_ videos. Our original aim was to prove only non-inferiority, but the evaluators judged the low-dose DVA videos slightly better (61 % preference, with 69 % interrater agreement), suggesting that the quality reserve of DVA was perhaps not fully used up by the applied low-dose protocol, and additional reduction might be possible. The elucidation of this question requires further studies.

The reduction of the amount of ICM in angiography procedures is of great importance for both patients and health care providers. As discussed in the introduction, there are numerous side effects of ICM that correlate with the volume applied [8]. By sparing half of the injected contrast agent without noticeable image quality loss, the ICM-associated procedural risk could be significantly lowered. The economic aspects are also substantial. CIN is the 3rd most common cause of hospital-acquired acute kidney injury [24]. It dramatically increases mortality, morbidity, length of stay and cost. The average extra cost is 10.345 $ in hospital and 11.812 $ in the 1st year [25]. Lowering the risk of CIN and CIE would obviously reduce the associated costs. Beyond these indirect savings, the direct cost of spared ICM is also considerable. The number of patients with significant carotid stenosis due to atherosclerosis is continuously rising, which generates a growing demand for minimally invasive procedures. The expected benefit is even higher if we take into consideration centres where non-invasive technologies are limited or not available, therefore intra-arterial X-ray angiography is used for both interventional and diagnostic examinations.

Our study has some limitations. The first and most important is the relatively low number of patients. Because of ethical considerations, we could not start with a large-scale trial, but rather with a proof-of-concept pilot study applying only a slight change in the local angiography protocol in accordance with the ethics committee approval. The protocol adjustment was based solely on the precisely measured quality reserve of DVA. The very convincing positive results provide a solid basis for a prospective multi-center study with a higher number of patients to further validate our findings.

The results and calculations presented in this paper are highly dependent on the local settings of the angiography system and the institutional standard-of-care protocol. It is important to emphasize that lab-specific comparative angiography procedures should be performed with DVA prior to any protocol modification in order to determine the amount of ICM dose reduction without image quality loss. The protocol of this study should not be applied directly in any protocol without precise adjustment to the local standard-of-care. It is also an open question whether the dilution of ICM or the reduction of the applied volume provides better results.

Our data suggest that refinement of the contrast injection protocol could result in further ICM dose reduction. The verification of this assumption requires further systematic research in the area. Further research is also needed to investigate how the quality reserve of DVA can be used to lower radiation dose. It is important to emphasize that using an optimized imaging protocol combined with a better suited angiography (e.g. a biplane) system is likely provides an even greater effect on contrast media and radiation reduction. Ongoing projects are aiming to further develop and validate DVA in different angiography systems. The elucidation of these questions has great clinical importance, since the possible reduction of ICM and radiation dose will obviously enhance the safety of minimally invasive endovascular interventions and diagnostic X-ray angiography.

## 5 Conclusions

DVA provides significantly higher SNR and better subjective image quality in carotid X-ray angiography than the current reference-standard DSA. This quality reserve of DVA allows a very substantial (50 %) ICM reduction without affecting the quality and diagnostic value of angiograms. Minimization of the amount of ICM improves the safety of the procedure by reducing the risk of adverse events, and provides significant economic advantage by reducing the direct cost of ICM and decreasing the cumulative cost of treatments following ICM-associated complications.

## Funding

The study was supported by the National Research, Development and Innovation Office of Hungary (NKFIA; NVKP-16-1-2016-0017 National Heart Program) and by the Thematic Excellence Program (2020-4.1.1.-TKP2020) of the Ministry of Innovation and Technology of Hungary, within the framework of the BIOImaging Excellence program at Semmelweis University.

## Ethical statement

This study was registered and approved by the National Institute of Pharmacy and Nutrition (reference number OGYÉI/69,206/2017) in Hungary. The protocol was designed in accordance with the standards of the Hungarian Medical Research Council and the Helsinki Declaration. All enrolled patients signed a written informed consent after being verbally informed by a physician.

## CRediT authorship contribution statement

**Viktor I. Óriás:** Conceptualization, Methodology, Investigation, Resources, Writing - original draft, Writing - review & editing, Project administration. **Dávid Szöllősi:** Methodology, Software, Formal analysis, Data curation, Writing - review & editing, Visualization. **Marcell Gyánó:** Conceptualization, Investigation, Writing - review & editing. **Dániel S. Veres:** Formal analysis, Data curation, Writing - review & editing. **Sándor Nardai:** Investigation, Writing - review & editing. **Csaba Csobay-Novák:** Investigation, Writing - review & editing. **Balázs Nemes:** Investigation, Writing - review & editing. **János P Kiss:** Formal analysis, Data curation, Writing - original draft, Writing - review & editing, Visualization. **Krisztián Szigeti:** Software, Resources, Writing - review & editing, Funding acquisition. **Szabolcs Osváth:** Software, Resources, Writing - review & editing, Funding acquisition. **Péter Sótonyi:** Conceptualization, Validation, Investigation, Writing - review & editing, Project administration. **Zoltán Ruzsa:** Conceptualization, Methodology, Validation, Investigation, Resources, Writing - review & editing, Supervision, Project administration.

## Declaration of Competing Interest

V.I.O., D.S and M.G. are part-time, J.P.K., K.S. and S.O. are full-time employees of Kinepict Health Ltd. K.S. and S.O. hold stocks of Kinepict Health Ltd, which is the manufacturer of the Kinepict Medical Imaging Tool. P.S. is a Scientific Advisor at Kinepict Health Ltd receiving no financial compensation for his work.
