# Supplementary material for: Initial evidence of a 50% reduction of contrast media using digital variance angiography in endovascular carotid interventions
Source: Eur J Radiol Open. 2020 Nov 17;7:100288. doi: 10.1016/j.ejro.2020.100288 (PMC7683322; doi:10.1016/j.ejro.2020.100288)
Supplement: Supplementary file 1 [file mmc1.docx]

**SUPPLEMENTARY MATERIAL**

The video comparison file contains three representative video pairs of DVA and DSA runs.

The corresponding numbers represent the same patient and the same acquisition direction. DVA videos were recorded with the low-ICM dose protocol (50 %, 3 ml ICM), whereas DSA videos were acquired with the standard-ICM dose protocol (100 %, 6 ml ICM). The DVA videos were generated with the cumulative technique, showing the still DVA image on the last frame. The brightness settings were adjusted automatically by the angiography system (DSA) or by the Kinepict Medical Imaging Tool software itself (DVA), the contrast settings were adjusted manually to set the same contrast level. To synchronize the videos the same frame number was taken from each run. Abbreviations: DSA: Digital Subtraction Angiography; DVA: Digital Variance Angiography; ICM: Iodinated Contrast Media. To see the videos, please start the file in presentation mode.
