# Supplementary figures and images for: Initial evidence of a 50% reduction of contrast media using digital variance angiography in endovascular carotid interventions
Source: Eur J Radiol Open. 2020 Nov 17;7:100288. doi: 10.1016/j.ejro.2020.100288 (PMC7683322; doi:10.1016/j.ejro.2020.100288)

## Slide 1
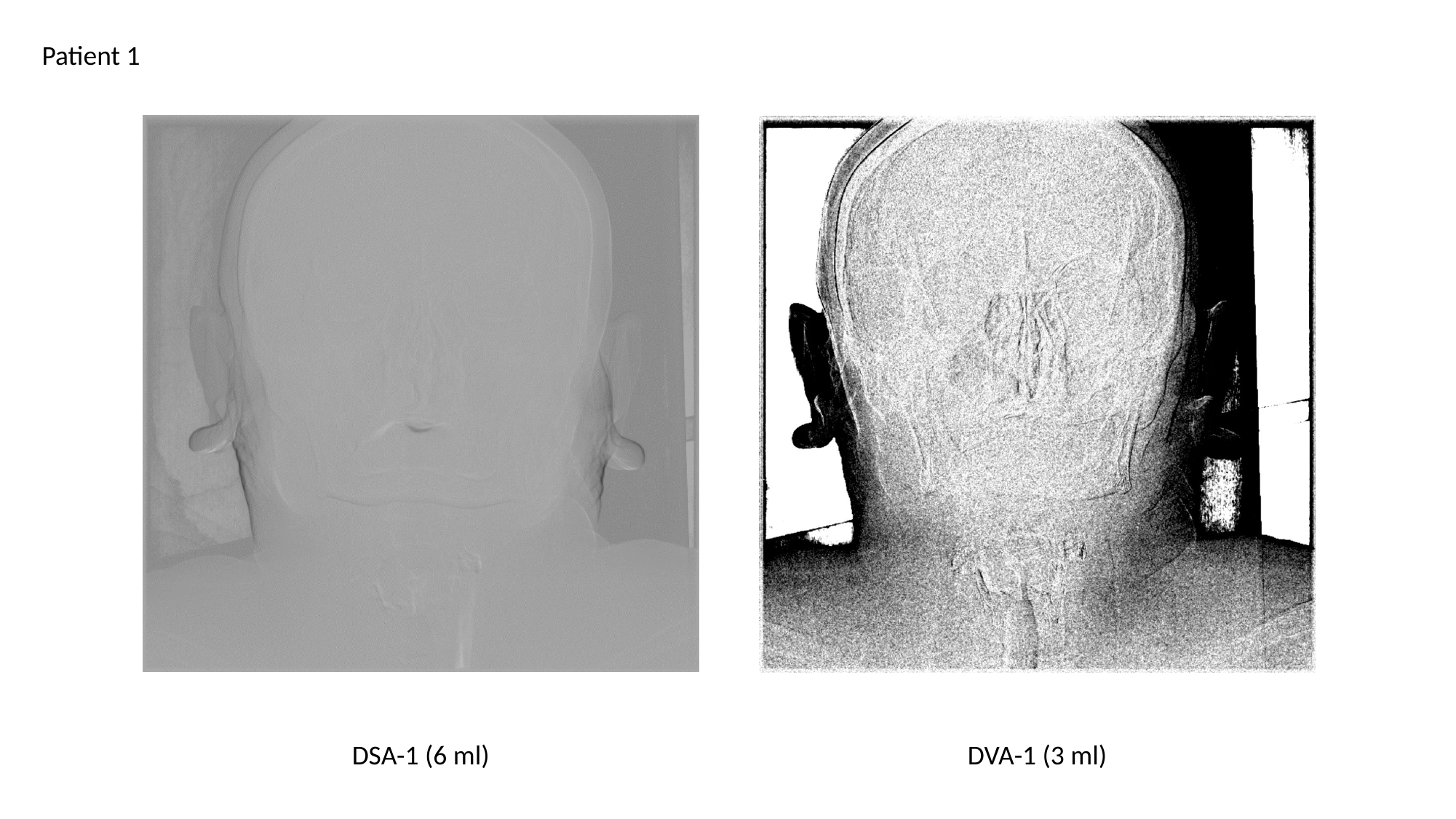

Patient 1
DSA-1 (6 ml)
DVA-1 (3 ml)

## Slide 2
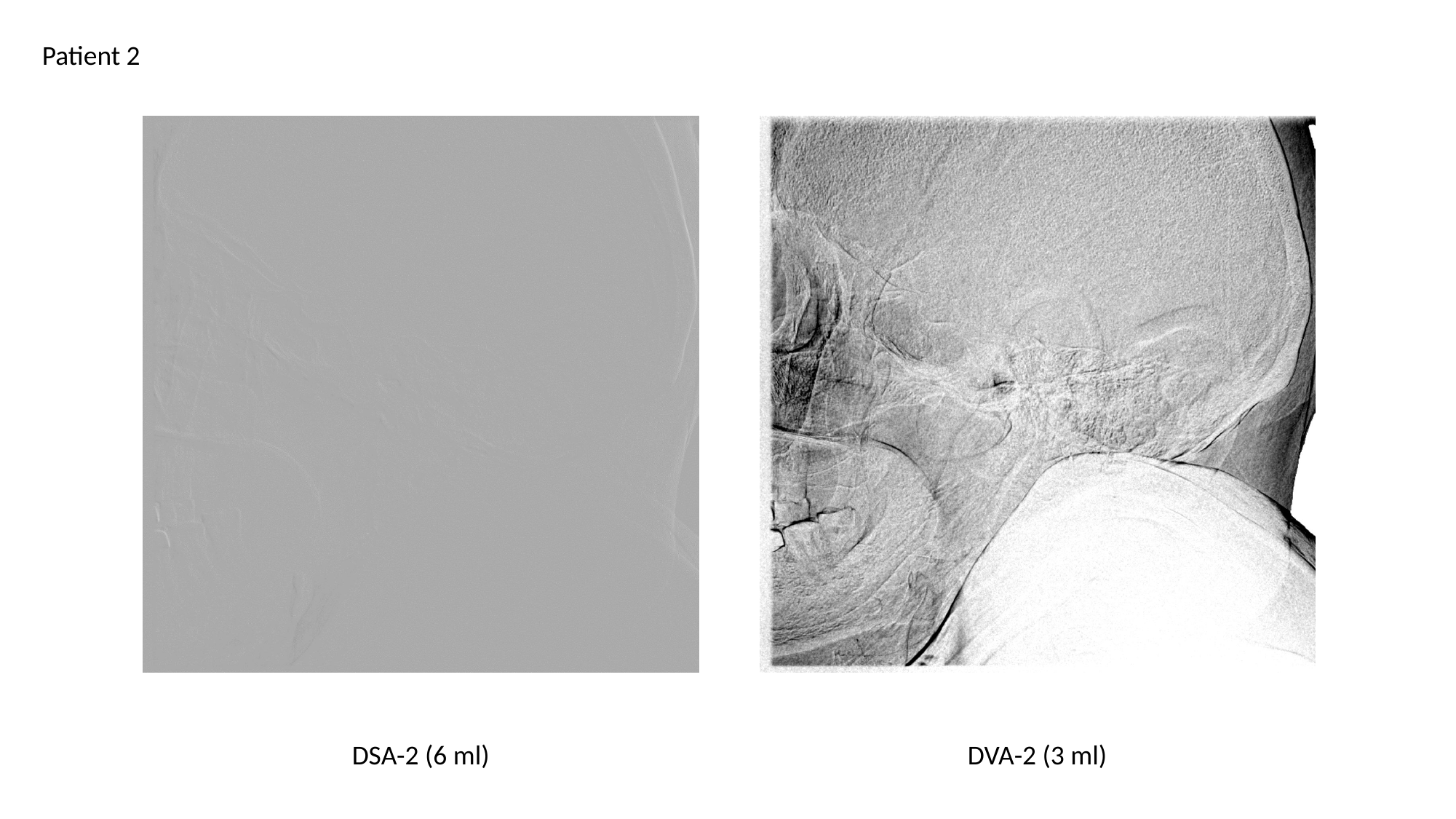

Patient 2
DSA-2 (6 ml)
DVA-2 (3 ml)

## Slide 3
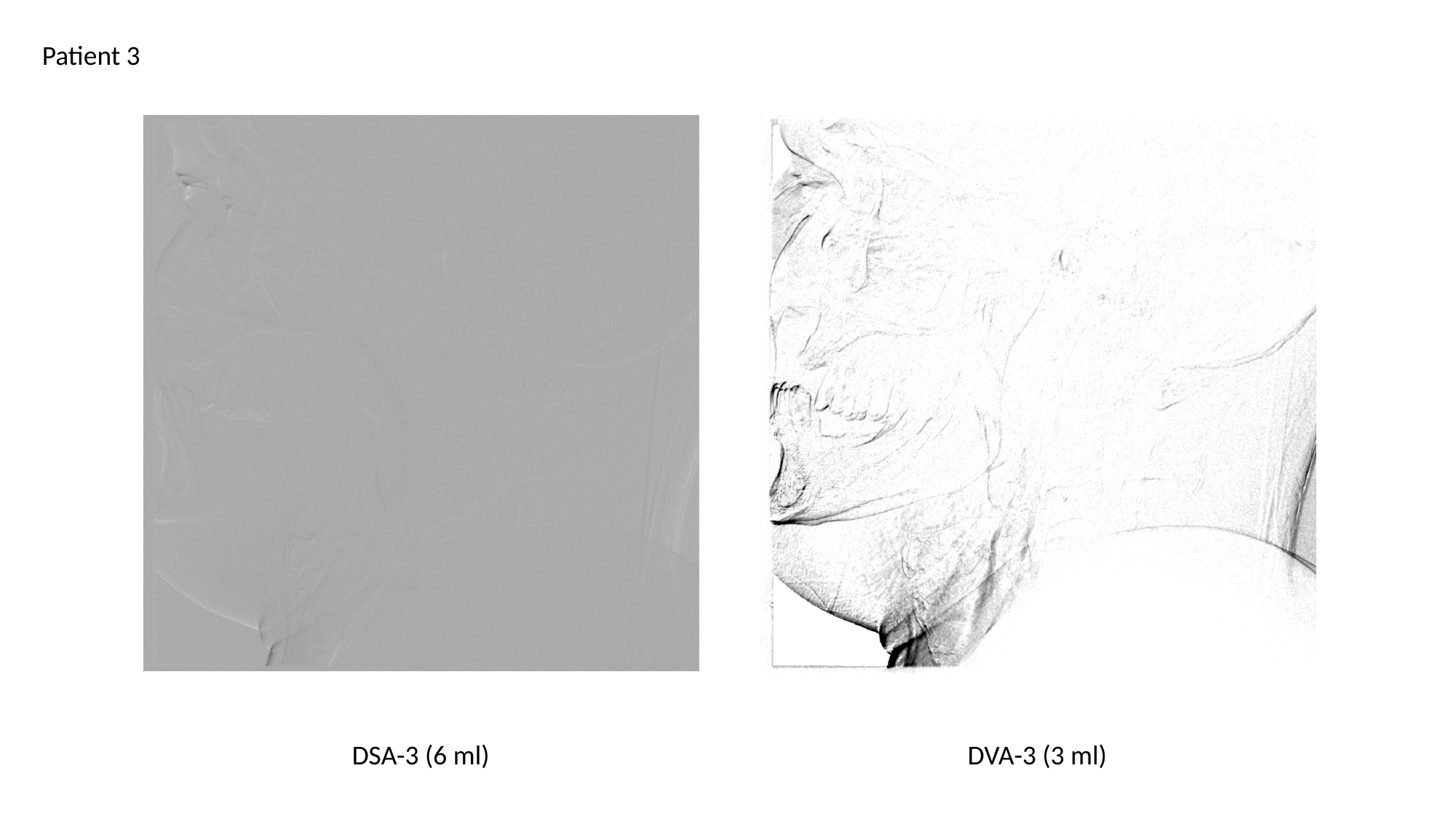

Patient 3
DSA-3 (6 ml)
DVA-3 (3 ml)

Supplement: Supplementary file 2 [file mmc2.pptx]
